# Effects of vulture exclusion on carrion consumption by facultative scavengers

**DOI:** 10.1002/ece3.3840

**Published:** 2018-02-01

**Authors:** Jacob E. Hill, Travis L. DeVault, James C. Beasley, Olin E. Rhodes, Jerrold L. Belant

**Affiliations:** ^1^ Carnivore Ecology Laboratory Forest and Wildlife Research Center Mississippi State University Mississippi State MS USA; ^2^ Animal and Plant Health Inspection Service Wildlife Services National Wildlife Research Center U.S. Department of Agriculture Sandusky OH USA; ^3^ Savannah River Ecology Laboratory University of Georgia Aiken SC USA; ^4^ Warnell School of Forestry and Natural Resources University of Georgia Athens GA USA; ^5^ Odum School of Ecology University of Georgia Athens GA USA

**Keywords:** *Cathartes aura*, competition, scavenging

## Abstract

Vultures provide an essential ecosystem service through removal of carrion, but globally, many populations are collapsing and several species are threatened with extinction. Widespread declines in vulture populations could increase the availability of carrion to other organisms, but the ways facultative scavengers might respond to this increase have not been thoroughly explored. We aimed to determine whether facultative scavengers increase carrion consumption in the absence of vulture competition and whether they are capable of functionally replacing vultures in the removal of carrion biomass from the landscape. We experimentally excluded 65 rabbit carcasses from vultures during daylight hours and placed an additional 65 carcasses that were accessible to vultures in forested habitat in South Carolina, USA during summer (June–August). We used motion‐activated cameras to compare carrion use by facultative scavenging species between the experimental and control carcasses. Scavenging by facultative scavengers did not increase in the absence of competition with vultures. We found no difference in scavenger presence between control carcasses and those from which vultures were excluded. Eighty percent of carcasses from which vultures were excluded were not scavenged by vertebrates, compared to 5% of carcasses that were accessible to vultures. At the end of the 7‐day trials, there was a 10.1‐fold increase in the number of experimental carcasses that were not fully scavenged compared to controls. Facultative scavengers did not functionally replace vultures during summer in our study. This finding may have been influenced by the time of the year in which the study took place, the duration of the trials, and the spacing of carcass sites. Our results suggest that under the warm and humid conditions of our study, facultative scavengers would not compensate for loss of vultures. Carcasses would persist longer in the environment and consumption of carrion would likely shift from vertebrates to decomposers. Such changes could have substantial implications for disease transmission, nutrient cycling, and ecosystem functioning.

## 1 INTRODUCTION

The geographic distribution of vultures (Families Cathartidae and Accipitridae, Subfamilies Aegypiinae and Gypaetinae) spans five continents, and throughout their range vultures fulfill an important ecological role through consumption of carrion (DeVault et al., 2016; Ogada, Keesing, & Virani, 2012). Scavenging can potentially reduce the spread of disease among wildlife because many pathogenic organisms on carcasses cannot survive passage through the highly acidic vulture digestive system (Beasley, Olson, & DeVault, 2015; Houston & Cooper, 1975). As the dominant consumers of carrion in many environments, vultures can indirectly impact other species because the presence of carrion influences the movement behavior of facultative scavengers and their prey (Cortés‐Avizanda, Selva, Carrete, & Donázar, 2009; Wilmers, Stahler, Crabtree, Smith, & Getz, 2003). Additionally, an absence of vultures can lead to increases in populations of facultative scavengers due to increased carrion availability (Markandya et al., 2008; Ogada, Keesing et al., 2012), and negative ecological impacts as some human commensals (e.g., rats and dogs) can be detrimental to native wildlife (Butler & du Toit, 2002; Young, Olson, Reading, Amgalanbaatar, & Berger, 2011).

The ecological functions performed by vultures often translate into direct benefits for humans (DeVault et al., 2016). Consumption of livestock carcasses by vultures precludes the need for people to pay for their removal (Margalida & Colomer, 2012). Vultures can also indirectly benefit humans through reduced risk of disease. For example, following the decline of vulture populations in India, populations of feral dogs increased, leading to an increase in cases of humans contracting rabies from feral dog bites (Markandya et al., 2008). The estimated health cost of this increase in rabies transmission from 1992 to 2006 was $34 billion (Markandya et al., 2008). Despite the benefits vultures can provide to people and the environment, vultures are the world's most threatened avian functional group (Şekercioğlu, 2006). Populations of vultures are experiencing continent‐wide declines in Asia and Africa due to threats such as poisoning, poaching, and collisions with power lines (Oaks et al., 2004; Ogada, Keesing et al., 2012; Ogada et al., 2016). Some populations have declined more than 90% in 20 years (Prakash et al., 2003) and 12 of the 22 species are now listed as endangered or critically endangered by the International Union for Conservation of Nature (Buechley & Şekercioğlu, 2016).

The ecological implications of such declines could be extensive because vultures consume a substantial amount of carrion. Vultures in Serengeti National Park, Tanzania consumed an estimated 14 million kilograms of meat annually, exceeding that of all mammalian carnivores combined (Houston, 1979). New World vultures in Central and South America may also consume more carrion than mammalian carnivores due to their proficiency at locating carrion in neotropical forests (Houston, 1994). Assuming a mean consumption rate of 0.3 kg/day of carrion for turkey vultures (*Cathartes aura*) (Chhangani, 2010; Singh & Chakravarthy, 2006) and a population size of 2 million in North America (Inzunza, Goodrich, & Hoffman, 2010), this species alone could potentially remove 219 million kg of carrion from the environment annually.

Carrion is abundant in most environments because many animals die from causes other than predation, making them potentially available as food for scavengers (Collins & Kays, 2011; DeVault, Rhodes, & Shivik, 2003). Anthropogenic activities such as collisions with automobiles or human‐made structures cause millions of animal deaths annually, further contributing to the amount of carrion available (Forman & Alexander, 1998; Loss, Will, & Marra, 2015). The removal of obligate avian scavengers and human‐induced increases in carrion results in considerable carrion availability that could subsidize populations of facultative mammalian scavengers (Markandya et al., 2008). Determining how such an increase in abundance of mammals might occur requires an understanding of the mechanisms influencing competition between vultures and mammals for carrion.

Competition between avian and mammalian scavengers is common at carcasses, and the outcome of these interactions depends on factors such as carcass detection ability (Houston, 1986; Shivik, 2006), habitat type (DeVault, Brisbin, & Rhodes, 2004; DeVault, Olson, Beasley, & Rhodes, 2011; Selva, Jędrzejewska, Jędrzejewska, & Wajrak, 2003; Turner, Abernethy, Conner, Rhodes, & Beasley, 2017), and scavenger body size (Butler & du Toit, 2002). Vultures frequently outcompete mammals for carrion through exploitation competition because flying enables vultures to traverse large areas more efficiently than mammals, often resulting in quicker detection times of carrion (Houston, 1979; Ruxton & Houston, 2004). This rapid detection can allow vultures to deplete carcasses before mammals can find them, with vultures consuming 90% of carcasses in some areas (Houston, 1986), although competition may change seasonally. Groups of avian scavengers can also monopolize carcasses and deter use by mammals. Scavenging ravens (*Corvus corax*) can deter wolves from carcasses (Vucetich, Peterson, & Waite, 2004), and Andean condors (*Vultur gryphus*) can cause pumas to abandon their kills (Elbroch & Wittmer, 2013).

Conversely, mammals dominate carrion consumption in some situations. In forested habitats where vultures have a decreased ability to detect carrion visually, mammals may consume more carcasses than vultures (Ogada, Torchin, Kinnaird, & Ezenwa, 2012; Turner et al., 2017). Nocturnal mammals also commonly deplete carcasses at night when avian scavengers are inactive (DeVault & Rhodes, 2002; Ogada, Torchin et al., 2012; Prior & Weatherhead, 1991). In Australia, for example, 88% of scavenging by mammals occurred at night (Huijbers, Schlacher, Schoeman, Weston, & Connolly, 2013). Mammal presence can prevent vultures from landing at carcasses (Prior & Weatherhead, 1991), and domestic dogs have used physical dominance to exclude vultures from carcasses (Butler & du Toit, 2002). In some habitats, the sheer abundance of mammalian carnivores results in mammals consuming most carrion (DeVault et al., 2011).

Ogada, Torchin, et al. (2012) demonstrated that when vultures were excluded from carcasses in Africa, there was an increase in the number of individual mammals using carcasses and the amount of time mammals spent at carcasses. There was also an increase in the number of contacts between mammals at carcasses in the absence of vultures, indicating an increased risk of disease transmission (Ogada, Torchin, et al., 2012). Considering the potential effects of vultures on the scavenging behavior of mammals and contact rates between individuals, there is a need to investigate these interactions in other ecosystems with different communities of avian and mammalian scavengers.

**Figure 1:**
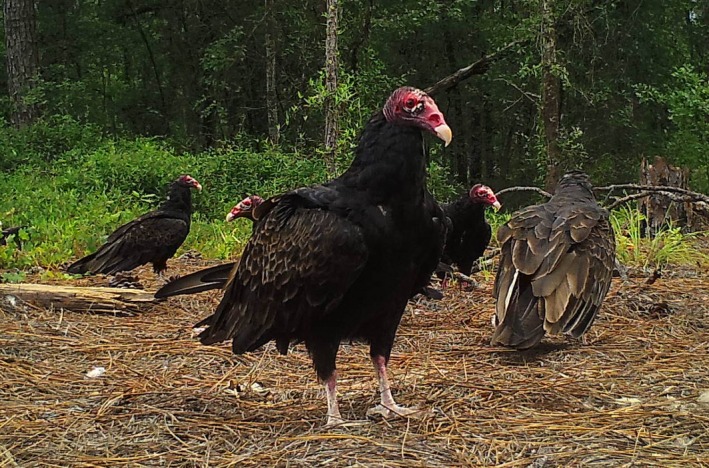
Turkey vultures (*Cathartes aura*) scavenging a rabbit carcass at the Savannah River Site, Aiken, SC

In North America, vulture diversity is primarily limited to turkey and black (*Coragyps atratus*) vultures (Figure 1), but numerous mammalian scavengers spanning several families are widely distributed (DeVault & Rhodes, 2002; DeVault, Brisbin et al., 2004; Turner et al., 2017). With divergent vulture and mammalian scavenging guilds among continents, it remains unclear to what extent vultures prevent mammals from consuming carrion in North America. Although black and turkey vultures are currently abundant in North America, it is possible that scavenging rates of mammals may increase should vulture populations decline and carrion availability subsequently increase, as has happened in India (Markandya et al., 2008). We explored competition for carrion between vultures and mammals by experimentally excluding vultures from carcasses to test the hypothesis that vultures outcompete mammalian scavengers for carrion through exploitation competition. Following Ogada, Torchin et al. (2012), we predicted that when vultures were excluded from carcasses, there would be (1) an increase in the presence of mammalian scavengers, (2) an increase in mammal species richness at carcasses, and (3) an increase in the persistence time of carcasses.

## 2 METHODS

### 2.1 Study site

We conducted this study at the Savannah River Site, a property owned by the US Department of Energy that encompasses 78,000 ha in Aiken County, South Carolina, USA (33°19′N, 81°42′W). The site is dominated by loblolly pine forests (*Pinus taeda*), longleaf pine forests (*Pinus palustris*), and bottomland hardwoods (e.g., *Nyssa* spp., *Quercus* spp.) (White & Gaines, 2000). Since 1951, much of the site has been managed for timber harvest and stands are harvested on a rotating basis (White & Gaines, 2000). We conducted this study during June–August 2016; average daily temperature was 27.6°C and average daily rainfall was 0.33 cm (NOAA 2017).

### 2.2 Study design

We selected 60 sites in pine (*Pinus* spp.) stands that were >20 years old that were within 15 m of a road. Choosing sites along roads facilitated accessing them twice daily (see below). Each site was separated by a minimum distance of 500 m. At these 60 sites, we conducted a total of 130 trials, randomly selecting 65 to serve as controls and excluding vultures from the remaining trials. We carried out 6 weeks of trials and each trial lasted 7 days. During each 7‐day period, we ran 20 trials (10 exclusion and 10 control) concurrently. We used separate sites in weeks 1–3 and reused these sites in the same sequence in weeks 4–6 (sites used in week 1 were reused in week 4, etc.). In the sixth and final week, we increased the number of trials to 30 (15 exclusion and 15 control) to redo trials that had failed due to camera malfunction. The 10 additional sites in the last week had also been used in the first and fourth weeks, so there was a minimum of 1 week between consecutive uses of the same site.

At each site, we placed a dark‐colored rabbit (*Sylvilagus* spp.) carcass weighing ~1,300 g that was obtained from a commercial supplier (RodentPro, Inglefield, IN, USA) and thawed to indoor ambient temperature. We used a cable lock to attach a motion‐activated infrared camera (Bushnell Trophy Cam HD Aggressor; Bushnell Corp., Overland Park, KS, USA) to a tree ~2 m from carcasses to record the presence of scavengers. Cameras took three pictures when motion‐activated, with a 1‐min delay between activations. To prevent scavengers from carrying carcasses beyond the field of view, we wrapped a nonrelaxing cable snare around each carcass and staked it to the ground with a 46‐cm steel rebar stake.

**Figure 2:**
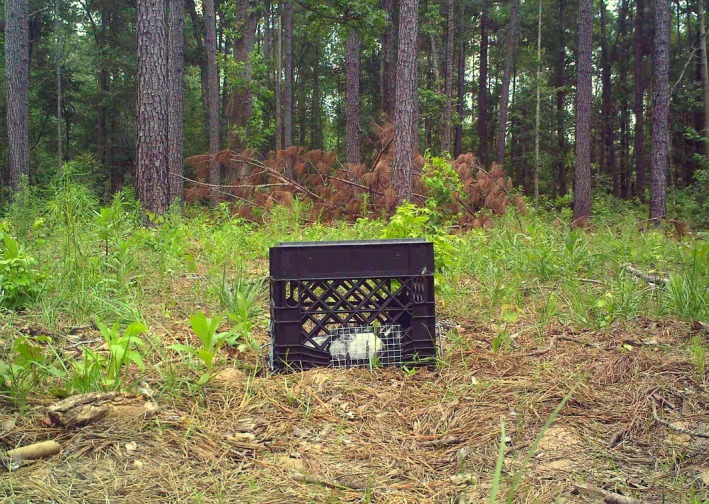
Placement of plastic crate (33.0 cm long, 33.0 cm wide, 27.6 cm tall) with panels of wire affixed over the handle openings over rabbit carcass to exclude diurnal scavenging. Logs were also placed along the perimeter to prevent vultures from reaching their bills under the edge of the crate and pulling out the carcass

To exclude vultures, we used a plastic crate that measured 33.0 cm long by 33.0 cm wide by 27.6 cm tall (Figure 2). We affixed panels of 1.27‐cm gauge wire mesh over the handle openings, so that vultures could not fit their heads into them. The crate had openings to permit airflow and access by arthropods, so that decomposition of exclusion carcasses would not differ from controls. As most mammalian scavengers at the site detect carrion by olfaction (DeVault & Rhodes, 2002), and the olfactory cues are produced by decomposers (DeVault et al., 2003), the openings in the crate minimized the chances that scavenger presence would be impacted by a difference in carcass detectability between the control and treatment trials.

To exclude vultures, which are diurnal, each day between 0700 and 1000 hr, we placed a crate on top of carcasses receiving the exclusion treatment. We used 30‐cm galvanized metal staples to secure the crate in place and placed logs around the perimeter to prevent vultures from reaching their bills under the crate. Crates were removed daily between 1800 and 2100 hr. Crates were only on carcasses during daylight hours, which prohibited diurnal scavenging. However, we believe this had minimal impact on scavenging rates by facultative scavengers as these species primarily scavenge at night (DeVault & Rhodes, 2002; DeVault et al., 2011; Huijbers et al., 2013). Previous research at SRS indicated that 91% of mammal visits to rabbit carcasses occurred between 1800 and 0900 hr (Turner et al., 2017).

Because our design excluded diurnal scavenging, it also incidentally excluded most facultative avian scavengers. However, visits to control carcasses by these species were rare, consisting of one visit each by an American crow (*Corvus brachyrhynchos*) and a red‐tailed hawk (*Buteo jamaicensis*) (see Results). In both cases, the bird was displaced by a vulture that consumed the majority of the carcass and scavenging by these species likely had a negligible impact on carcass consumption. We removed these species from our analysis because they could not access the exclusion carcasses, but maintained these two trials in analysis. We visited control carcasses twice daily to standardize human presence between the treatment and control trials. For each carcass, we documented the date when there appeared to be no edible flesh remaining on the carcass and considered the carcass fully scavenged at that time.

From the photographs, we identified scavenger species at each carcass and examined whether nonavian scavengers were present. Results are expressed as the number of carcasses at which the species was present. We compared the presence/absence of all nonavian scavengers combined between the treatment and control using a generalized linear model with binomial distribution and logit link using R version 3.2.3 (R Core Team, 2016). We also calculated species richness of nonavian scavengers at each carcass and compared this variable between the control and treatment using a generalized linear model with a quasi‐Poisson distribution (to account for overdispersion of data) and log link. To compare the carcass detection time, we calculated time between carcass placement and when an animal was first observed at the carcass for vultures at control carcasses, mammals at control carcasses, and mammals at exclusion carcasses. Treatments were compared using a generalized linear model with normal distribution and identity link. We used the Kaplan–Meier procedure to compare the time to carcass depletion between the treatment and control using the R package “survival” (Therneau, 2015). We chose this procedure because there was a single binary predictor. We right‐censored trials in which the carcass had not been fully consumed at the end of 7 days. A *p*‐value of .05 was used to determine statistical significance for all analyses.

## 3 RESULTS

Of the 130 trials, 110 produced usable data (53 control and 57 exclusion). We censored trials due to camera failure (*n *=* *15) and failure to prevent vultures from accessing exclusion carcasses (*n *=* *4). The latter happened when vultures arrived at the carcass when the crate was absent or when vultures were able to pull the carcass from under the crate and consume it. We also censored one exclusion trial when the carcass was consumed by a red‐tailed hawk while the crate was not positioned on the carcass. At exclusion sites, there were 122 detections of mammals (i.e., a mammal in at least one of the three pictures taken when the camera was triggered, including multiple detections of the same species at a carcass and those that did not scavenge) at night when the crate was not positioned over the carcass and only two detections during daylight when the crate was over the carcass. Thus, our use of crates during daylight hours effectively excluded vultures while only minimally impacting carcass accessibility by mammals.

Turkey and black vultures scavenged at 50 and 10 control carcasses, respectively. Mammals recorded scavenging at control carcasses were coyote (*Canis latrans*,* n *=* *1), Virginia opossum (*Didelphis virginiana*,* n *=* *2), and wild pig (*Sus scrofa*,* n *=* *1). Scavengers recorded at exclusion carcasses (at night when crates were removed) were coyote (*n *=* *3), opossum (*n *=* *6), wild pig (*n *=* *1), and American alligator (*Alligator mississippiensis*,* n *=* *2). More than one species was detected at 13 carcasses (Table 1). Facultative scavengers scavenged at 9% of control carcasses and 19% of exclusion carcasses. Fifty control carcasses were consumed by scavengers and three were not scavenged. By contrast, only 11 exclusion carcasses were scavenged, whereas 46 were not scavenged.

**Table 1:** Presence of vertebrate scavengers consuming rabbit carcasses at the Savannah River Site, Aiken SC (June–August 2016)

Treatment	Number of trials	Turkey vulture	Black vulture	Coyote	Opossum	Wild pig	American alligator
Control	38	Χ					
	8	Χ	Χ				
	1	Χ		Χ			
	1	Χ	Χ			Χ	
	1	X			X		
	1	X	X		X		
Exclusion	5				Χ		
	3			Χ			
	2						Χ
	1				Χ	Χ	

**Figure 3:**
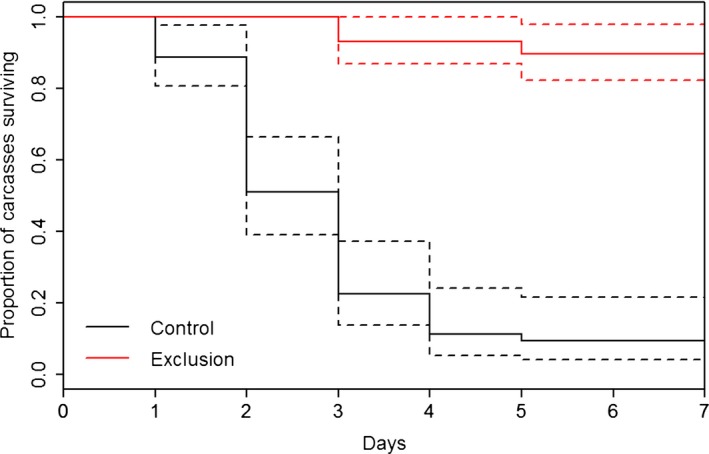
Days to complete rabbit carcass consumption by vertebrate scavengers at the Savannah River Site, Aiken SC (June–August 2016) between carcasses from which vultures were excluded and controls, estimated using the Kaplan–Meier procedure. Dashed lines represent 95% confidence intervals

**Figure 4:**
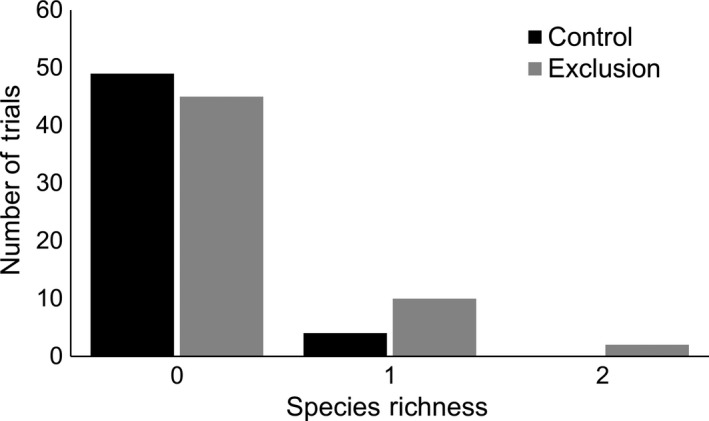
Species richness of nonavian scavenger species that visited rabbit carcasses from which vultures were excluded (*n *=* *57) and controls (*n *=* *53) at the Savannah River Site, Aiken SC (June–August 2016). Generalized linear model with quasi‐Poisson distribution and log link indicated no difference in occurrence of nonavian species richness between the exclusion and control carcasses (β = .6204, *p*‐value = .203)

Vultures arrived at control carcasses on average 1.96 ± 0.83 days after placement. Mammals arrived at exclusion carcasses on average 3.02 ± 2.34 days after placement and at control carcasses on average 3.20 ± 1.91 days after placement. Vultures at control carcasses arrived sooner than mammals at exclusion carcasses (β = −1.0755, *p*‐value = .004). Control carcasses were scavenged more quickly than exclusion carcasses (χ^2^ = 86.3, *p*‐value < .001, Figure 3). Compared to control carcasses, there was a 1.1‐ and 8.5‐fold increase in the percentage of available exclusion carcasses at the end of 2 and 4 days, respectively. At the end of the trials (7 days), there was a 10.1‐fold increase in the number of available exclusion carcasses compared to control carcasses. Treatment was not a significant predictor of nonavian scavenger presence (β = 1.0748, *p*‐value = .083) or nonavian scavenger species richness (β = .6204, *p*‐value = .203, Figure 4).

## 4 DISCUSSION

Mammals did not scavenge more frequently in the absence of vulture competition, and we found no support for our hypothesis that vultures would outcompete mammals for carrion through exploitation competition. Similarly, our prediction that there would be an increase in the presence and species richness of nonavian scavengers when vultures were excluded was not supported by our findings. However, the predicted increase in carcass persistence did occur because when vultures could not access a carcass, it was unlikely to be scavenged by vertebrates.

The increase in carcass persistence indicates that under the environmental conditions in this study, mammals were unable to functionally replace vultures as scavengers. In Spain, ungulate carcasses persisted longer in areas without vultures (Morales‐Reyes et al., 2017) and Ogada, Torchin et al. (2012) also documented an increase in ungulate carcass persistence when vultures were experimentally excluded in Africa. Facultative scavengers may not be able to compensate for the loss of dominant scavengers even when the dominant scavengers are not vultures. Facultative avian scavengers consume most carrion in Australia, and fish carcasses lasted longer in urban areas with lower avian scavenger abundance (Huijbers et al., 2015). In an agricultural landscape where raccoons (*Procyon lotor*) were the dominant scavenger (DeVault et al., 2011), rodent carcasses persisted longer when raccoons were removed (Olson, Beasley, DeVault, & Rhodes, 2012). Scavenging by mammals increased following reductions in dominant scavenger abundance in each of these studies, but not at a high enough rate to remove carcasses as efficiently as the dominant scavengers.

A notable difference in our study was that there was not a significant increase in mammal scavenging when vultures were excluded, and thus not even partial compensation of the loss of scavenging by vultures. This pattern was likely influenced by season, as we conducted this study in summer, when the average daily temperature was 27.6°C and maximum temperature exceeded 32.2°C on most days (NOAA 2017). Microbial activity generally increases with warmer temperatures (Pechal et al., 2013; Putman, 1978), and bacteria can produce noxious chemicals that deter scavenging by animals when they colonize carcasses (Burkepile et al., 2006; Janzen, 1977). This increase in decomposer activity can decrease the time that carcasses are palatable to mammals and mammals generally scavenge less during warmer temperatures (e.g., DeVault, Brisbin et al., 2004; Selva, Jędrzejewska, Jędrzejewski, & Wajrak, 2005; Turner et al., 2017). Vultures may be more tolerant than mammals to toxins produced by decomposers, making carcasses available to them for a longer period of time than they are to mammals (Chung et al., 2015; Houston & Cooper, 1975; Roggenbuck et al., 2014).

Invertebrate decomposers are also more active during warmer temperatures and can rapidly consume carcasses. At another location in South Carolina, arthropods began to reduce the mass of pig carcasses weighing 1,000–1,400 g after 2 days and reduced the body mass of carcasses by 90% within 6 days (Payne, 1965). Because vultures typically arrived <2 days after placement (and sometimes within 1 day), there likely had not been substantial carrion consumption by invertebrates when they detected carcasses. However, invertebrate consumption may have increased considerably by the time that mammals arrived, which was on average more than 1 day later. When environmental conditions facilitate rapid decomposition of carcasses, the ability of vultures to quickly detect carrion likely makes them more efficient scavengers than mammals and might partially account for the inability of mammals to replace vultures as the dominant scavengers under these conditions. As the majority of exclusion carcasses were not consumed at the end of the 7‐day trials, it is possible that mammals may have scavenged carcasses after monitoring ended. However, the advanced state of decomposition of carcasses after 7 days makes it unlikely that they would have been scavenged by mammals (Payne, 1965).

Another factor contributing to the lack of scavenging by mammals could be that for some facultative scavengers, carrion is a resource consumed primarily when other resources are scarce (Jędrzejewski & Jędrzejewska, 1992; Jędrzejewski, Zalewski, & Jędrzejewska, 1993; Read & Wilson, 2004). At SRS, coyotes predominately consume vegetation such as blackberries (*Rubus* spp.) and wild plums (*Prunus* spp.) in summer and shift to mammalian food items in winter as vegetative food items become scarcer (Schrecengost, Kilgo, Mallard, Ray, & Miller, 2008). The abundance of vegetative food items in summer may lead coyotes to consume less carrion during this time because other foods are available. Similarly, the diet of opossums in summer consists largely of vegetation, but may switch to carrion in the winter when other resources become scarce (Hopkins, 1974). For both species, we documented instances in which individuals were present at carcasses before vultures arrived. Thus, scavenging by mammals at our study site was not solely dependent on the ability to detect carcasses, but is likely also influenced by the availability of alternative food.

Seasonality can influence vertebrate scavenging at SRS, with a decrease in vulture activity and increase in mammal scavenging during winter (Turner et al., 2017). Therefore, mammals may compete more effectively with vultures during cooler seasons and might functionally replace vultures in the removal of carrion under such conditions. However, temperatures are warm for much of the year at this location and mean monthly temperature typically exceeds 21.1°C for 5 months or more each year (NOAA 2017). Furthermore, annual temperature in the region is projected to increase 2.2–2.5°C in the next 50 years (Kunkel et al., 2013). Thus, even if mammals are capable of replacing vultures in carrion removal during cooler seasons, were vultures to become extirpated from this area, there would still be a substantial portion of the year in which carrion would mostly not be scavenged by vertebrates.

The degree to which vulture presence influences species richness of mammalian scavengers can vary, either by increasing species richness by alerting other scavengers to the presence of carrion (Sebastián‐González et al., 2016), or by decreasing species richness by exploiting the resource before other scavengers are able to detect it (Ogada, Keesing et al., 2012; Ogada, Torchin et al., 2012). The low species richness of nonavian scavengers in our study can be attributed in part to the use of rabbit carcasses, as smaller carcasses generally support fewer scavenger species (Moleón, Sánchez‐Zapata, Sebastián‐González, & Owen‐Smith, 2015). Vulture presence did not influence scavenger species richness in our study because mammals scavenged infrequently regardless of competition with vultures. There were a few instances in which mammals scavenged on control carcasses after vultures had scavenged it partially. The evisceration of these carcasses may have facilitated mammal detections of carrion by making it more detectable through olfaction, but there was not a large enough sample size to test this. Although most studies on such facilitative effects of scavenger species focused on visual cues provided by vultures to mammals (e.g., Kane & Kendall, 2017; Sebastián‐González et al., 2016), they may also provide olfactory cues when carcasses are not completely consumed.

An important aspect of our study is that vultures were present, but excluded from scavenging our trial carcasses. This contrasts with studies such as Morales‐Reyes et al. (2017) in which vultures were entirely absent from the study area; this difference could be meaningful for facultative scavengers. Although vultures could not scavenge experimental carcasses, they were abundant on the site and thus scavenging on other carrion sources, reducing the total availability of carrion in the area. If vultures were absent altogether, carrion availability would likely increase substantially. As facultative scavengers may switch from predation to scavenging as carrion becomes more available (Van Dijk et al., 2008), a true absence of vultures may lead to increased mammal scavenging due to increased selection of carrion compared to live prey. We were unable to examine such potential shifts in foraging behavior. Also, detection ability is a major factor influencing scavenging behavior under the environmental conditions of our study (Turner et al., 2017); we are uncertain whether mammals would be able to increase their detection times of carrion enough to substantially increase carrion consumption if vultures were truly absent from the study area.

The spacing of carcasses could have also influenced scavenger detections. Distance between sites was based on the availability of sites that met our habitat requirements, and our minimum distance of 500 m between sites could have resulted in spatial dependence in terms of scavenger detection of carcasses. However, the overall infrequent detections of mammals, especially within any set of 20 trials, suggest that the same individuals did not scavenge multiple carcasses as a result of carcass proximity. Additionally, our overall mean detection time at control carcasses of 1.96 days was similar to that of 2.20 days reported in another study of scavenging of rabbit carcasses at SRS during summer (Turner et al., 2017). Therefore, we suggest that the spacing of carcasses did not have a substantial impact on scavenger behavior.

Despite these limitations, our study suggests that a decline in vultures in our study area would likely result in a shift in the cycling of nutrients through food webs. Because mammals are not likely to increase carrion consumption in the absence of competition with vultures, at least during summer months, consumption of this resource would shift from vertebrates to decomposers. This shift could promote increased prevalence of disease‐causing bacteria, such as *Mycobacterium bovis*, which are known to colonize several species of mammal carcasses (Gortázar et al., 2008; Naranjo, Gortazar, Vicente, & De La Fuente, 2008). Some arthropods such as blowflies (Diptera: Calliphoridae) that use carrion can also carry diseases (Maldonado & Centeno, 2003). However, some toxic bacteria may not survive the digestive tracts of blowflies (Mumcuoglu, Miller, Mumcuoglu, Friger, & Tarshis, 2001), so disease‐causing decomposers on carcasses may impose some controls on each other. How the overall presence of these decomposers would be impacted by an increase in carrion remains unclear. Most studies of the role of carrion in disease transmission have used ungulate carcasses (e.g., Bellan, Turnbull, Beyer, & Getz, 2013; Gortázar et al., 2008; Jennelle, Samuel, Nolden, & Berkley, 2009), and the potential for toxic microbes on smaller mammal carcasses such as those in this study has been less explored.

The spatial distribution of nutrients provided by carcasses would also be impacted by vulture declines. Nutrients from carcasses are distributed throughout the landscape by vultures, which generally have large home ranges because they are obligate scavengers (Beasley et al., 2015; DeVault, Reinhart, Brisbin, & Rhodes, 2004; Ruxton & Houston, 2004). Had they scavenged extensively, coyotes might have had a similar impact on nutrient dispersion, as they are known to cache food items (e.g., Windberg, Knowlton, Ebbert, & Kelly, 1997) and have an average home range size of 31.85 km^2^ at SRS (Schrecengost, Kilgo, Ray, & Miller, 2009). However, the lack of scavenging we documented on control carcasses indicates that instead of being dispersed throughout the landscape, nutrients would remain spatially clustered near the carcasses (Melis et al., 2007). Nutrients from carcasses can enter the soil, augmenting plant growth (Bump et al., 2009). The clustering of nutrients around a carcass due to a lack of vertebrate scavenging may impact surrounding plant communities and by extension the organisms that consume those plants (Carter, Yellowlees, & Tibbett, 2007). Although most studies have focused on how vulture declines impact other scavengers, (e.g., Kane & Kendall, 2017; Morales‐Reyes et al., 2017; Ogada, Torchin et al., 2012), our results indicate that the ecological impacts of vulture loss could extend to lower trophic levels as well.

## CONFLICT OF INTEREST

None declared.

## AUTHOR CONTRIBUTIONS

All authors conceived the idea and designed the methodology. JEH collected the data. JEH and JLB analyzed the data. JEH led the writing of the manuscript. All authors contributed critically to the drafts and gave final approval for publication.

## DATA ACCESSIBILITY

Data available from the Dryad Digital Repository: https://doi.org/10.5061/dryad.6qd19.
